# MicroRNA-15b is induced with E2F-controlled genes in HPV-related cancer

**DOI:** 10.1038/bjc.2011.457

**Published:** 2011-11-01

**Authors:** M P Myklebust, O Bruland, Ø Fluge, A Skarstein, L Balteskard, O Dahl

**Affiliations:** 1 Section of Oncology, Institute of Medicine, University of Bergen, Bergen 5021, Norway; 2 Center of Medical Genetics and Molecular Medicine, Haukeland University Hospital, Bergen 5021, Norway; 3 Department of Oncology and Medical Physics, Haukeland University Hospital, Bergen 5021, Norway; 4 Department of Surgical Sciences, University of Bergen, Bergen 5021, Norway; 5 Department of Surgery, Haukeland University Hospital, Bergen 5021, Norway; 6 Department of Oncology, University Hospital of Northern Norway, Tromsø 9038, Norway

**Keywords:** miR-15b, anal carcinoma, HPV, E2F

## Abstract

**Background:**

MicroRNAs (miRNAs) are important regulators of cellular processes and are found to be deregulated in many cancers. We here analysed the miRNA expression in anal carcinomas. In a previous study, we found that our anal carcinoma tumours were divided into two groups based on the expression of E2F-regulated genes. Therefore, we searched for miRNAs that could reproduce this grouping.

**Methods:**

A global screen of the miRNA population was performed using real-time quantitative PCR (RT–qPCR) array methods and differentially expressed miRNAs were identified. Real-time–qPCR was used to verify the expression levels of selected miRNAs and genes in a larger collection of biopsies. A siRNA-mediated knockdown of human papilloma virus (HPV)16 E7 in a cervical cell line was performed to assess the effect of E7 on miR-15b.

**Results:**

The grouping of tumours into two groups based on the expression of E2F-controlled genes was confirmed in a larger collection of anal carcinoma tumours. The expression of miR-15b was shown to be highly correlated with that of five selected E2F-induced genes (*CCNA2*, *CCNB1*, *CCNB2*, *MSH6* and *MCM7*). A knockdown of HPV16 E7 resulted in decreased levels of miR-15b in Ca Ski cells.

**Conclusion:**

MiR-15b expression correlates with E2F-regulated genes in anal carcinoma and appears to be part of the E2F-regulatory network.

The expression of microRNAs (miRNAs) are altered in a number of human carcinomas and in many physiological processes in the organism (Calin *et al*, 2002; Michael *et al*, 2003; van Rooij *et al*, 2006; Volinia *et al*, 2006; Sonkoly *et al*, 2007; Wang *et al*, 2008). MicroRNAs are a class of conserved, short, non-coding RNAs that modulate gene expression through the RNAi pathway (Hutvagner and Zamore, 2002; Bartel, 2004). Mature miRNAs are single-stranded ∼22 nucleotides derived from hairpin-structured miRNA precursors, by the RNaseIII enzymes Drosha and Dicer (Lee *et al*, 2002; Bartel, 2004). MicroRNAs differ from siRNAs in that they generally require only partial complementarity to the target sequence, which is often in the 3′-untranslated region of the mRNAs. Binding of a miRNA to a target mRNA sequence leads to transcriptional or post-transcriptional changes of the expression of the corresponding protein (Bartel, 2004; Lim *et al*, 2005; Rajewsky, 2006). Cleavage of the target mRNA may occur when complementarity is perfect. This occurs mostly in plants, but can also be seen in animals (Hutvagner and Zamore, 2002; Zeng and Cullen, 2003). MicroRNAs have been postulated to regulate ∼30% of the human genome (Bartel, 2004).

In cancers, miRNAs were proposed to work either as tumour suppressors or as oncogenes through regulation of the protein expression that contributes to oncogenesis (Esquela-Kerscher and Slack, 2006). Through genome-wide mapping of known miRNAs in the human genome, miRNA genes were located to fragile sites and to other genomic regions often involved in cancer development (Calin *et al*, 2004). For example, the relative incidence of miRNAs at human papilloma virus (HPV)16 integration sites was significantly higher than in the rest of the genome. This is interesting as HPV is accepted as an aetiological agent of some cancers, such as cervical cancer and anal squamous carcinoma (zur Hausen, 1989; Frisch *et al*, 1997; Daling *et al*, 2004; Madsen *et al*, 2008). Squamous cell carcinoma of the anal canal is a rare type of cancer with increasing incidence, probably related to changes in sexual behaviour (Daling *et al*, 2004). HIV-positive patients also have increased risk of developing anal cancer due to immune suppression secondary to HIV infection (Grulich *et al*, 2007). The transforming capability of HPV is assigned to E6 and E7 oncogenes (zur Hausen, 2000). These two proteins inactivate p53 and the retinoblastoma (RB1) protein, respectively (Dyson *et al*, 1989; Werness *et al*, 1990). Hence, two important pathways in cells that regulate cell growth and apoptosis are inhibited, leading to uncontrolled cell growth. Retinoblastoma protein 1 is a negative regulator of E2F transcription factors, which among others induce the transcription of many genes that control cell fate and proliferation, such as *MCM7*, *MSH6*, *cyclin E*, *cyclin A* and *cyclin B* (Iaquinta and Lees, 2007). Recently, HPV E7 was also reported to actually modulate the miR-203 level in keratinocytes to maintain cells in the proliferative state to produce virus particles (Melar-New and Laimins, 2010).

In a previous study, we demonstrated that tumours from patients with anal carcinomas could be divided into two distinct groups based on global mRNA expression (Bruland *et al*, 2008). All tumours expressed HPV16 E7 mRNA. Cluster analysis on a number of E2F and/or high-risk HPV-regulated genes reproduced these two groups. Thus, HPV16 had major influences on the global gene expression in approximately half of the tumours studied. In this study, the aim was to study global miRNA expression in anal carcinomas. Therefore, the patient database and the biopsy material from the previous study have been supplemented with 11 additional tumours. The grouping of tumours into two groups based on E2F and/or high-risk HPV-regulated gene expression was verified in the larger number of HPV-positive anal carcinoma tumours. Here, we report that one miRNA was found to divide tumours into the same two groups, namely miR-15b.

## Materials and methods

### Biopsies and tissue

In all, 24 biopsy samples from 18 different patients with anal carcinoma and 4 mucosa biopsy samples from different patients with benign anorectal disorders (benign polyps) were obtained at the Haukeland University Hospital in Bergen and at the University Hospital of Northern Norway in Tromsø (Norway). Tissue samples were directly snap frozen in liquid nitrogen and stored at −80 °C. The diagnoses were verified by a histopathologist and classified according to the TNM stages. Cytological smears obtained from the biopsies immediately before tissue lysis and RNA extraction were evaluated to ensure representativity, that is, the presence of tumour cells.

### RNA preparation

Total RNA was extracted from the snap-frozen biopsies using the RNeasy mini-kit, Spin Technology (Qiagen GmbH, Hilden, Germany) according to the manufacturer's protocol. In all, 3*μ*g of total RNA was subjected to DNase treatment to remove contaminating DNA (TURBO DNase no. 2338, Ambion, Austin, TX, USA). Single nucleotides were excluded using the RNA clean-up protocol with spin columns (RNeasy, Qiagen GmbH). Total RNA including small RNAs from cell cultures was extracted using miRNeasy mini kit (Qiagen GmbH). The quantity and quality of total RNA were assessed using OD 260/280 (NanoDrop Technologies, Wilmington, DE, USA) and the Experion Automated Electrophoresis System (Bio-Rad, Irvine, CA, USA). Total RNA from a non-tumour anal biopsy (NT11) and a pool of RNA from three other anal biopsies with no malignancies (NT8-10) were prepared for aiding in the interpreting of real-time (RT)–PCR results.

### Quantitative RT–PCR analysis

To quantify mRNA expression of the genes of interest, cDNA synthesis was performed as described by the manufacturer using the high-capacity cDNA reverse transcription kit including Random Hexamers (P/N 4374966, Applied Biosystems, Foster City, CA, USA). The following TaqMan assays (Applied Biosystems) were used: MCM7 (assay ID Hs00428518m1), MSH6 (Hs00264721_m1), CCNB1 (Hs00259126_m1), CCNB2 (Hs00270424_m1), CCNA2 (Hs00153138_m1) and ACTB (Hs99999903_m1). Primers and probes for detection of HPV E7 were obtained from Sigma-Aldrich (St. Louis, MO, USA): HPV16 forward primer 5′-CCGGACAGAGCCCATTACAAT-3′, reverse primer 5′-ACGTGTGTGCTTTGTACGCAC-3′, probe 5′(FAM)-TGTTGCAAGTGTGACTCTACGCTTCGGT-(TAMRA) 3′. HPV18 (forward primer 5′-GACTCAGAGGAAGAAAACGATGAAA-3′, reverse primer 5′-GTGACGTTGTGGTTCGGCT-3′, probe 5′-(FAM)-TGGAGTTAATCAT CAACATTTACCA-(TAMRA)-3′ (Lamarcq *et al*, 2002).

A high-throughput screen of the global miRNA expression was performed using multiplex primer pools (Applied Biosystems) and TaqMan Array Human MicroRNA Panel, Early Access version (P/N 4384792, Applied Biosystems). Each 10 *μ*l multiplex reaction contained 25 ng DNase-treated RNA template. For more detailed description on materials and methods used in global miRNA analysis, please see Supplementary Data, Section 1. Individual TaqMan MicroRNA Assays (Applied Biosystems) were performed on the complete set of RNA samples on selected miRNA assays to verify the results of the Human MicroRNA panels. Assays analysed were hsa-miR-15b (P/N 4373122), hsa-miR-203 (P/N 4373095), hsa-miR-let7b (P/N 4373168), hsa-miR-151 (P/N 4373179), hsa-miR-365 (P/N 4373194), hsa-miR-221 (P/N 4373077), hsa-miR-572 (P/N 4381017), hsa-miR-147 (P/N 4373131) and hsa-let-7d (P/N 4373166). Reverse transcription was carried out using the TaqMan MicroRNA reverse transcription kit (P/N 4366596) according to the manufacturer's instructions. See Supplementary Data, Section 2, for more details on materials and methods on the individual miRNA analyses. Each reaction contained 15 ng DNase-treated RNA template.

The relative difference of mRNA and miRNA expression was calculated using the 2^-ΔΔCt^ method (Livak and Schmittgen, 2001). Endogenous controls were *β*-actin and RNU48 for RNA and miRNA analyses, respectively, as these were stably expressed in all our samples. The expression was calculated relative to the pool of three non-tumour anal biopsies (NT8-10). To calculate the relative expression of HPV16 E7 between samples, the median of the E2F-based subgroup of patients with lowest HPV16 load (group 1) was used as a calibrator as the NT samples were HPV16 negative. The software J-Express Pro version 2.7 (UiB, Bergen, Norway) (Dysvik and Jonassen, 2001) was used for visualising the expression results and cluster analysis.

### Cell line and transient transfection

Two siRNAs were designed for the silencing of HPV E7 mRNA, si198 targeting nt 773–791 (sense 5′ → 3′GCACACACGUAGACAUUCG) (Tang *et al*, 2006) and si752 targeting nt 754–774 (sense 5′ → 3′CUUCGGUUGUGCGUACAAAGC) (Yamato *et al*, 2008) of the HPV16 genome (GenBank accession no. NC_001526). Negative control no. 1 (Ambion P/N 4635), a non-targeting siRNA sequence, was used as negative transfection control, named ‘siCtrl’ from here.

To test the effect of miR-15b on cyclin E, transient transfection hsa-miR-15b inhibitor (AM10904) with anti-miR mock control no. 1 (AM17012) and hsa-miR-15b precursor (Ambion, P/N PM10904) with precursor mock control no. 1 (AM17121) were performed in the Ca Ski cell line.

The cell line used in these experiments was the HPV16-positive cervical epithelial carcinoma Ca Ski cell line (CRL-1550, ATCC-LGC, Teddington, UK). Ca Ski cells are of epithelial morphology and derived from an epidermoid carcinoma metastasis located in the small intestine, primary tumour located in cervix. The HPV16 viral load is ∼600 copies per cell. All incubations were carried out in a humified atmosphere and 5% CO_2_ at 37 °C. Cells were maintained in RPMI-1640 with stable L-glutamine (E15-885, PAA GmbH, Pasching, Austria), 10% FBS (A15-101, PAA) and penicillin–streptomycin (P11-10, PAA). The reverse transfection protocol recommended by Ambion was used for all transfections. In brief, the appropriate siRNA, miR-15b inhibitor or miR-15b precursor at a final concentration of 30 nM was incubated using NeoFX transfecting agent (AM no. 4510, Ambion) in serum-reduced OptiMem (P/N 51985-026, Invitrogen, Carlsbad, CA, USA) and overlaid with cells in growth medium. After 48 h, cells were trypsinised, washed and harvested. One fraction submitted to RNA extraction, one fraction to cell counting and cell phase analysis and one to protein analysis. The transfections were performed in triplicate, and each experiment was repeated at least once.

### Cell-cycle analysis

Cells transfected with siRNAs, precursor miR-15b and miR-15b inhibitor or controls as described, were trypsinised, washed with PBS and subjected to cell-cycle analysis using NucleoCounter NC-3000 (Chemometec, Allerød , Denmark). The analysis method was a two-step cell-cycle analysis in which cell lysis and staining of the nuclei with DAPI are performed.

### Western blot analysis

Cells were lysed in RIPA buffer containing 25 mM Tris-HCl pH 7.6, 150 mM NaCl, 1% IgePal 60, 1% sodiumdeoxycholate, 0.1% SDS and complete EDTA-free protease inhibitor (Roche Applied Science, Indianapolis, IN, USA). The proteins were separated by electrophoresis in a NuPAGE 4–12% bis-Tris gradient gel (Invitrogen). After gel electrophoresis, proteins were electroblotted to a nitrocellulose membrane. After blocking, the membrane was incubated with an antibody specific for HPV16 E7 (sc-51951, Santa Cruz Biotechnology, Santa Cruz, CA, USA), *β*-actin (no. 4967, Cell Signaling, Beverly, MA, USA), cyclin B1 (sc-752), cyclin A (sc-751), MCM7 (ab2360, Abcam, Cambridge, UK), cyclin E1 (C4976, Sigma-Aldrich) or GAPDH (sc-25778, Santa Cruz Biotechnology). After washing with TBST, the membrane was incubated for 1 h at room temperature with HRP-labelled secondary antibody (sc-2005 or sc-2004, Santa Cruz Biotechnology). The results were visualised by SuperSignal West Pico Chemiluminescent Substrate (Pierce Biotechnology, Rockford, IL, USA). *β*-Actin or GAPDH were used as loading control.

### Statistics and ethics

To test whether the observed differences in the expression of the tested mRNAs, miRNAs and percentages in cell-cycle phases were statistically significant, the results were analysed with a two-tailed Student's *t*-test (*P*=0.05). Correlation was tested using Pearson's correlation (*P*=0.01). In the correlation analyses, values for each of the tumours in the two groups were compared. The SPSS 18.0 statistical package was used for *t*-tests and correlation testing of the RT– qPCR results (SPSS Inc., Chicago, IL, USA). Graphs were produced using the GraphPad Prism version 4.00 software for Windows (GraphPad Software, San Diego, CA, USA).

Patients gave written, informed consent to sampling of a separate biopsy for research purpose. This study was approved by the Regional Ethics Committee and thus complies with regulations for clinical research in Norway.

## Results

### Clustering of the biopsies

**Figure 1:**
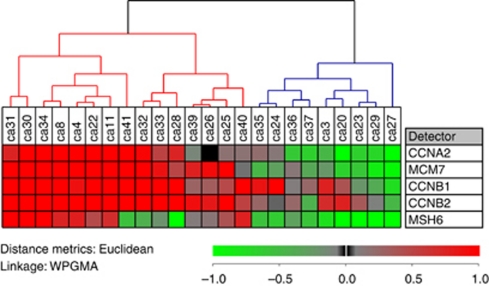
Classification of tumour biopsies into two groups based on the mRNA expression profile of five E2F-controlled genes. The expression levels were obtained by RT–qPCR and normalised to the non-tumour mucosa samples, that is, expressions of these genes in tumours are shown relative to expressions in non-tumour samples. Group 1 has a blue dendrogram and Group 2 a red.

**Figure 2:**
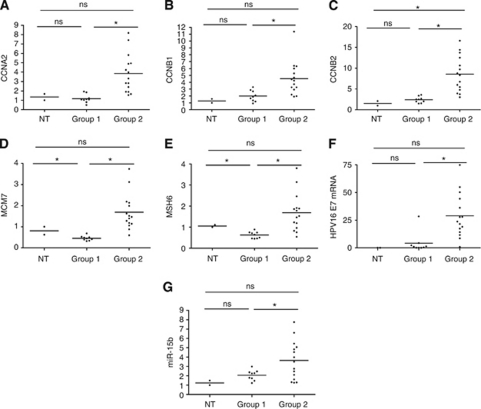
Graphs showing the distribution of mRNA expression of the five E2F-regulated genes (**A**–**E**), HPV16 E7 (**F**) and miR-15b (**G**) of the anal carcinoma biopsies. Expression was assayed by RT–qPCR and calculated relative to non-tumour mucosas (NT8-10). The means of the groups are indicated. Two-sided Student's *t*-test was used to compare the means, and *P*-values were considered statistically significant when <0.05. ^*^P<0.05. ns=not significant.

Unsupervised hierarchical cluster based on relative quantification of the 5 E2F-induced genes, *CCNA2*, *CCNB1*, *CCNB2*, *MSH6* and *MCM7*, by RT–qPCR divided the 24 tumours into two groups. The expressions of these genes were generally higher in group 2 than in group 1 (Figures 1 and 2).

### Identification of HPV16 and HPV18 virus mRNA

Quantitative PCR was used to test tumour samples for HPV16 and HPV18 E7 mRNA. Of the 24 tumour biopsies, 23 tested positive for HPV16 and 1 tested positive for HPV18 (ca3). One biopsy expressed both HPV16 and HPV18 mRNA (ca4). The biopsies from non-malignant anal mucosa were HPV16 and HPV18 E7 negative. The amount of HPV16 E7 mRNA was generally higher in group 2 than in group 1 (Figure 2F, *P*<0.001).

### MiR-15b is differently expressed in tumours

The TaqMan MicroRNA array provided a screening of the expression levels of 365 miRNAs. In all, 10 miRNAs initially reproduced the E2F and/or high-risk HPV-regulated genes subgroups (data not shown). After single-assay RT–qPCR verification analysis, only miR-15b showed statistically significant differences in expression levels between the two groups. Group 2 has higher expression of miR-15b than does group 1 (*P*=0.012) and the biopsies from non-malignant mucosa (*P*=0.080). Thus, miR-15b is upregulated in group 2, which also has upregulated expression of several E2F-regulated genes (Figure 2). This result indicates that there is a statistic significant correlation between E2F gene regulation and miR-15b expression in our tumours (Table 1). The levels of HPV16 E7 mRNA correlate well with both the mRNA levels of five well-established E2F-regulated genes (*CCNA2*, *CCNB1*, *CCNB2*, *MCM7* and *MSH6*) and miR-15b. The *P*-values for correlations were generated by comparing mRNA values from each tumour using Pearson's correlation.

MiR-203 has been proposed to be suppressed by HPV E7 in keratinocytes to keep the cells in a proliferating state (Melar-New and Laimins, 2010). One of the targets of miR-203 is the p63 protein family. In our anal biopsies, we could not detect a statistically significant difference in the expression of miR-203 between non-tumour biopsies and tumour biopsies, or between the two tumour groups based on the results from RT–qPCR. The initial miRNA analysis in a subset of samples described above, identified miR-203 as a candidate, but further analysis revealed no significant differentiated expression in group 1 and group 2. No difference in TAp63 and ΔpN63*α* mRNA level was detected between non-tumour and tumour biopsies or between tumour groups either (data not shown).

### Knockdown of HPV16 E7 in cell culture affects miR-15b expression

**Figure 3:**
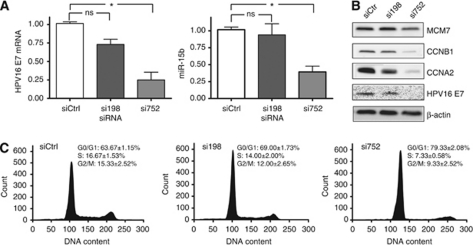
(**A**) Relative amounts of HPV16 E7 mRNA and miR-15b in Ca Ski cells 48 h after transfection of mock siRNA (siCtrl) and two different siRNAs targeting HPV16 E7, si198 and si752. The relative expression levels were obtained by RT–qPCR. Endogenous controls were *β*-actin for HPV16 E7 and RNU48 for miR-15b. (**B**) Effect of HPV16 E7 knockdown on protein levels of E2F-regulated genes. Immunodetection of cyclin B1, cyclin A2, MCM7 and HPV16 E7 in whole-cell lysates from Ca Ski cells 48 h after transfection. *β*-Actin was used as loading control. (**C**) Effects of HPV16 E7 knockdown on cell-cycle distribution in Ca Ski cells. The plots shown are distribution profiles representative for the replicates. Values shown are mean percentages of cells in each phase with s.d. ^*^P<0.05, statistically significant difference between control samples and knockdown samples. ns=not significant.

Knockdown of HPV16 E7 in Ca Ski cell cultures was performed using two different siRNAs, namely si198 and si752. The relative expression of HPV16 E7 mRNA in control and knockdown samples were 1.01±0.02 for controls, 0.73±0.12 for si198 and 0.25±0.18 for si752 (Figure 3A). Analysis by western blot confirmed that changes seen at the mRNA level were reflected at the protein level (Figure 3B). Reduced amount of E7-mRNA yielded reduced amount of the E7 protein in knockdown samples, compared with control samples. Cyclin A, cyclin B1 and MCM7 proteins were all reduced in HPV16 E7 knockdown samples confirming that proliferation had decreased as expected. The miR-15b expression was examined by RT–qPCR in lysates from cell cultures and found to be downregulated in knockdown samples (Figure 3A). The relative expression of miR-15b for siCtrl, si198 and si752 was 1.02±0.07, 0.94±0.16 and 0.39±0.09, respectively. Knockdown of HPV16 E7 thus resulted in a downregulation of miR-15b proportional to the decrease in E7 in the Ca Ski cell line.

The percentage of cells in different stages of the cell cycle was different in E7 knockdown samples compared with controls (Figure 3C). Our data showed that cells transfected with siRNA against HPV16 E7 had a higher percentage of cells in the G_0_/G_1_ phase and a lower percentage of cells in the S and G_2_/M phases. This indicates that the G_0_/G_1_ check point was restored after E7 knockdown. The decrease in percentages was proportional to the level of E7 knockdown. For G_0_/G_1_, the differences between both siCtrl and si198, that between and siCtrl and si752, were statistically significant (*P*=0.011 and *P*<0.001, respectively). For the S phase and G_2_/M phase, only the difference between siCtrl and si752 was significant (*P*=0.004 and *P*=0.043, respectively). si752 yielded a more effective knockdown of E7 than did si198, and the effects on miR-15b expression and cell-cycle progression were more pronounced in si752 samples than in si198 samples.

### Effects of miR-15b on cyclin E

**Figure 4:**
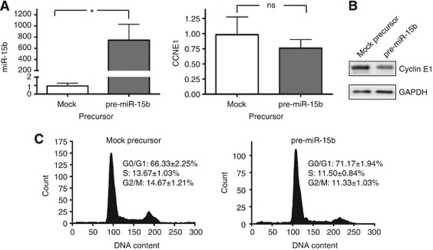
(**A**) Relative amounts of miR-15b and cyclinE1 mRNA in Ca Ski cells 48 h after transfection of mock precursor (precursor Ctrl no. 1) and precursor miR-15b. The relative expression levels were obtained by RT–qPCR. RNU48 and *β*-actin were used as endogenous controls, respectively. (**B**) Immunodetection of cyclin E1 in whole-cell lysates 48 h after transfection of precursor miR-15b into Ca Ski cells. GAPDH was used as loading control. (**C**) Effects of transfection of precursor miR-15b on cell-cycle distribution in Ca Ski cells. The distribution profiles shown are representative for the replicates. Values shown are mean percentages of cells in each phase with s.d. ^*^P<0.05, statistically significant difference between control samples and knockdown samples. ns=not significant.

To test the effect of upregulation or downregulation of miR-15b on endogenous cyclin E1 in the HPV-positive Ca Ski cell line, transient transfections of commercially available precursor miR-15b and anti-miR-15b were performed. The relative amount of mature miR-15b after transfection of precursor was 0.96±0.32 for the mock precursor and 744±287 for the miR-15b precursor measured by RT–qPCR (Figure 4A). A slight decrease in cyclin E1 mRNA levels was detected after upregulation of miR-15b, but the difference was not statistically significant (Figure 4A). Immunodetection of cyclin E1 showed a modest decrease at the protein level after transfection of miR-15b precursor (Figure 4B). For the anti-miR15b, we were unable to achieve a reproducible knockdown of miR-15b in this cell line. The cyclin E1 protein levels were not altered in anti-miR15b-transfected samples compared with controls (results not shown).

Transfection of precursor miR-15b into Ca Ski cells resulted in an increase of the amount of cells in the G_0_/G_1_ phase (Figure 4C), and the percentages of cells in the S phase and the G_2_/M phase were reduced. These differences in cell-cycle distribution between mock precursor and miR-15b precursor-transfected cells were statistically significant (*P*=0.003, *P*=0.003 and *P*<0.001, respectively). As for the knockdown of HPV16 E7, transfection of precursor miR-15b seemed to restore the G1/S check point.

## Discussion

Differences in miRNA profiles between normal cells and cancer cells have been identified in several cancers. The changes in the genome that leads to cancer, for example, mutations, deletions, insertions or amplifications, may result in alternations of the miRNA profile. It is now known that the expressions of miRNAs are transcriptionally regulated similar to protein-coding genes, for example, by transcription factors like E2F and c-MYC. MiRNAs may act as oncogenes or tumour suppressors depending on their target. Initially, we searched for miRNAs that were differently expressed in anal tumours with higher *vs* lower expression of E2F-induced genes. These two groups were identified among the anal tumours in a previous study (Bruland *et al*, 2008). In this former study, we identified some of the anal tumours to be more influenced by E2F-inducable promoters, and in this study, we have shown that the mir-15b expression correlated well with the mRNA expression of the studied E2F-induced genes in each of the individual tumours. The E2F family of transcription factors is an important regulator of the cell cycle, and is used by the HPV to take control of proliferation in the infected cell. The E7 protein of HPV binds to pRB, a negative regulator of E2F, and disrupts its binding to E2F (Dyson *et al*, 1989). The HPV E6 protein inactivates the tumour-suppressor protein p53 (Werness *et al*, 1990). Taken together, these two events lead to the dysfunctional G1/S check point and impaired response to DNA damage, prerequisites for genomic instability.

To investigate whether the elevated miR-15b levels in group 2 of anal carcinomas were actually associated with HPV infection, we transfected a HPV16-positive cell line with siRNA against E7. By knocking down HPV16 E7, we inhibited the activity of E2F, and thereby the expression levels of E2F-inducible genes were reduced. Ca Ski cells are HPV-positive SCC of epithelial-type cell originating from the cervix; thus, we chose this cervical cell line for our experiment as no anal carcinoma cell line was commercially available. Knockdown of E7 resulted in an increased fraction of cells in the G_0_/G_1_ phase of the cell cycle. This is expected when the G_0/_G_1_ check point is restored, offering a robust measure of E7 inhibition. Ca Ski showed decreased levels of miR-15b after knockdown of E7, indicating that the observed elevated miR-15b in group 2 is associated with HPV infection and thus indirectly, E2F. Upregulation of miR-15b in the same cell line by transfection of the precursor miR-15b, led to an increase of cells in the G_0_/G0 phase, but the proportions of cells in the S and G_2_/M phases were decreased. This indicates that miR-15b has an inhibitory effect on cell-cycle progression. Although the mRNA levels of cyclin E1 was not statistically reduced after upregulation of miR-15b in the Ca Ski cell line, the levels of cyclin E1 protein were reduced. This may be due to the miRNAs function as post-transcriptional modulators (Pillai *et al*, 2005; Chekulaeva and Filipowicz, 2009).

**Figure 5:**
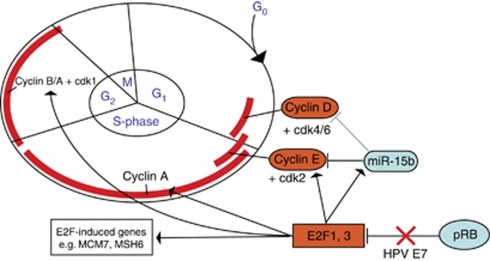
A schematic model for the putative role of miR-15b in the cell cycle-regulatory network controlling the transition of cells from the G1 phase to the S phase. Bar-headed lines represent inhibition, whereas arrows indicated stimulation. Human papilloma virus (HPV) abolishes the binding of pRB by activating E2F-transcription factors. Faint, grey lines represent predicted interactions. Factors that stimulate the cell cycle are red, inhibitors are blue.

These findings are in concordance with findings from others. Bueno *et al* (2010) showed that miR-15b is induced by E2F transcriptions factors in mouse embryonic fibroblasts, E2F1 and E2F3 in particular. Binding sites for E2F1 and E2F3 were identified in the promoter of miR-15b. This finding supports both our findings and our working hypothesis that miR-15b is regulated by E2F transcription factors. Recently, another group showed that miR-15 and miR-16 are transcriptionally regulated by E2F1 and targets cyclin E in a feed-forward loop (Ofir *et al*, 2011). In glioma cells, cyclin E1 was identified as a direct target of miR15b through transfection studies (Xia *et al*, 2009). Cyclin D1 was by computational methods predicted to be a downstream target of miR-15b. Our finding that miR-15b correlated with other E2F-regulated genes in anal carcinoma tumours, adds support to cell line experiments linking miR-15b and E2F. Others have published similar results that link miR-15b and its miRNA family to cell-cycle regulation (Linsley *et al*, 2007; Liu *et al*, 2008; Bueno *et al*, 2010)). These findings place miR-15b in the cell proliferation and cell survival network. Cyclin D and E together with cdk4/6 and cdk2 regulate the progression through the G_1_ check point from the G_0_/G_1_ to the S phase. Hence, miR-15b may act as a negative feedback regulator by inhibiting cyclin E1 and possibly D1, and thus inhibit enhanced cell proliferation and prevent replicative stress triggered by increased mitogenic stimulation for example, HPV infection (illustrated in Figure 5).

In summary, we have identified the expression of miR-15b as strongly associated with the expression of several E2F-regulated genes in anal carcinoma biopsies. The expression of miR-15b is reduced after HPV16 E7 knockdown in the cervical SCC cell line, Ca Ski. We have also showed that an upregulation of miR-15b in this cell line leads to a downregulation of cyclin E1 protein.

## Figures and Tables

**Table 1:** Pearson's correlation analysis between miR-15b expression and E2F-regulated genes

**Parameter**	**CCNA2**	**CCNB1**	**CCNB2**	**MCM7**	**MSH6**	**HPV16 E7**
R	0.570	0.766	0.591	0.499	0.634	0.557
Significant? (*α*=0.01)	Yes	Yes	Yes	Yes	Yes	Yes
*P*-value (two-tailed)	0.002	*P*<0.001	0.001	0.009	0.001	0.003
