# Supplementary material for: MicroRNA-15b is induced with E2F-controlled genes in HPV-related cancer
Source: Br J Cancer. 2011 Nov 1;105(11):1719–25. doi: 10.1038/bjc.2011.457 (PMC3242611; doi:10.1038/bjc.2011.457)
Supplement: Supplementary Information [file bjc2011457x1.doc]

## Supplementary data

### Detailed material and methods for RT-qPCR miRNA

#### Quantitative real-time PCR, global miRNA analysis

A high through-put screen of the global miRNA-expression was performed on nine biopsies from anal carcinomas and from four non-tumour (NT) anal mucosas. RNA from three of the non-tumour samples (NT8, NT9 and NT10) were pooled and used as one sample. The main reason for pooling of NT8-10 was low RNA-contents. Multiplex primer-pools from Applied Biosystems were used for preparing cDNA for miRNA analysis on TaqMan® Array Human MicroRNA Panel, Early Access-version (P/N 4384792, Applied Biosystems). Reverse transcription reactions were prepared using the TaqMan MicroRNA Reverse Transcription Kit (P/N 4366596, Applied Biosystems) according to the manufacturer’s protocol. Each 10 µl multiplex reaction contained 25 ng RNA template. Prior to real-time PCR, the multiplex RT-reactions were diluted 62.5 fold with nuclease-free water. The diluted RT-products were mixed with TaqMan 2X Universal PCR Master Mix, No AmpErase® UNG (P/N 4324018, Applied Biosystems) and loaded on the TaqMan® Array Human MicroRNA Panel.

### Quantitative real-time PCR, analysis of individual miRNAs

Individual TaqMan® MicroRNA Assays (Applied Biosystems) were performed on the complete set of RNA samples on selected miRNA assays to verify the results of the Human MicroRNA Panels. Assays analysed were hsa-miR-15b (P/N 4373122), hsa-miR-203 (P/N 4373095), hsa-miR-let7b (P/N 4373168), hsa-miR-151 (P/N 4373179), hsa-miR-365 (P/N 4373194), hsa-miR-221 (P/N 4373077), hsa-miR-572 (P/N 4381017), hsa-miR-147 (P/N 4373131) and hsa-let-7d (P/N 4373166). Reverse transcription was carried out using the TaqMan® MicroRNA Reverse Transcription Kit (P/N 4366596) according to the manufacturer’s instructions Each reaction contained 15 ng DNase-treated RNA as template.. Real-time PCR was performed using TaqMan Universal PCR Master Mix, No AmpErase UNG (P/N 4324018, Applied Biosystems) and TaqMan® Assay miRNA Mix (Applied Biosystems) as recommend by the manufacturer.
